# The Reliability of Neuromuscular and Perceptual Measures Used to Profile Recovery, and the Time-Course of Such Responses Following Academy Rugby League Match-Play

**DOI:** 10.3390/sports8050073

**Published:** 2020-05-22

**Authors:** Hendrickus G. J. Aben, Samuel P. Hills, Darren Higgins, Carlton B. Cooke, Danielle Davis, Ben Jones, Mark Russell

**Affiliations:** 1 School of Social and Health Sciences, Leeds Trinity University, Leeds LS18 5HD, UK; h.aben@leedstrinity.ac.uk (H.G.J.A.); s.hills@leedstrinity.ac.uk (S.P.H.); c.cooke@leedstrinity.ac.uk (C.B.C.); d.davis@leedstrinity.ac.uk (D.D.); 2 Castleford Tigers RLFC, The Mend-A-Hose Jungle, Castleford WF10 2SD, UK; darrenhiggins@castigers.com; 3 Carnegie Applied Rugby Research (CARR) Centre, Leeds Beckett University, Leeds LS6 3QT, UK; b.jones@leedsbeckett.ac.uk; 4 England Performance Unit, The Rugby Football League, Leeds LS17 8NB, UK; 5 Leeds Rhinos Rugby Club, Leeds LS6 3BR, UK; 6 School of Science and Technology, University of New England; Armidale, NSW 2351, Australia; 7 Division of Exercise Science and Sports Medicine, Department of Human Biology, Faculty of Health Sciences, the University of Cape Town and the Sports Science Institute of South Africa, Cape Town 7725, South Africa

**Keywords:** fatigue, recovery, muscle damage, team sport, isometric mid-thigh pull, countermovement jump, wellness, athlete monitoring

## Abstract

In professional academy rugby league (RL) players, this two-part study examined; (A) the within- and between-day reliability of isometric mid-thigh pulls (IMTP), countermovement jumps (CMJ), and a wellness questionnaire (*n* = 11), and (B) profiled the responses with acceptable reliability (no between-trial differences and between-day coefficient of variation (CV) ≤10% and intraclass correlation coefficient (ICC) ≥0.8) for 120 h (baseline: −3, +24, +48, +72, +96, +120 h) following RL match-play (*n* = 10). In part A, force at 200, and 250 ms, and peak force (PF) demonstrated acceptable within- (CV%: 3.67–8.41%, ICC: 0.89–0.93) and between-day (CV%: 4.34–8.62%, ICC: 0.87–0.92) reliability for IMTP. Most CMJ variables demonstrated acceptable within-day reliability (CV%: 3.03–7.34%, ICC: 0.82–0.98), but only six (i.e., flight-time, PF, peak power (PP), relative PP, velocity at take-off (VTO), jump-height (JH)) showed acceptable between-day reliability (CV%: 2.56–6.79%, ICC: 0.83–0.91). Only total wellness demonstrated acceptable between-day reliability (CV%: 7.05%, ICC: 0.90) from the questionnaire. In part B, reductions of 4.75% and 9.23% (vs. baseline; 2.54 m∙s^−1^; 0.33 m) occurred at +24 h for CMJ VTO, and JH, respectively. Acceptable reliability was observed in some, but not all, variables and the magnitude and time-course of post-match responses were test and variable specific. Practitioners should therefore be mindful of the influence that the choice of recovery monitoring tool may have upon the practical interpretation of the data.

## 1. Introduction

Rugby league is a team sport characterised by high-intensity activities such as high-speed (≥5.5 m∙s^−1^) running and sprinting (≥7.0 m∙s^−1^) actions that are interspersed with contact efforts (i.e., collisions, wrestling and grappling), and low-intensity activities such as standing, walking and jogging [1,2,3]. Largely due to the frequency and intensity of eccentric muscle actions and physical contacts [4,5], the demands of match-play may cause post-match perturbations in the hormonal milieu [5,6], indices of neuromuscular function [4,7,8], perceptual responses [5,9], and muscle soreness [4]. Knowing the influence of match-play on specific recovery and preparedness to train markers is valuable for practitioners when seeking to modulate training intensity and/or volume thereafter in order to avoid the accumulation of fatigue and subsequent injury, illness and/or underperformance [10].

Up to 120 h may be required to facilitate full post-match recovery [8], however most observations from adult players have reported durations of 48–72 h [7,11] when profiling the restoration of neuromuscular, biochemical or endocrine, and/or perceptual responses [12]. These inconsistencies may reflect methodological differences between studies, such as the reliability of the specific variables being examined [13], between-study differences in match-play demands, as well as discrepancies in training regimes [8,14] and recovery strategies [7,11] implemented in the post-match period; all of which are known to modulate post-match recovery [12]. Literature reporting the reliability of the various recovery markers used in collision-sports players is limited, in both senior [15], and academy [13] playing standards. Furthermore, whilst some investigations have reported reliability data, it is unclear whether these relate to within- or between-day assessments [5,6]. Such information may be important, especially when considering the repeated use of certain measurements in either within- or between-day scenarios. As the reliability of measures may be population-specific [15], it is important for practitioners to know the reproducibility of tests and variables in their target population.

Previous studies that have profiled post-match responses in rugby league players have often recruited senior age players [4,5,7,9], and typically neglect those in the later stages of adolescence (i.e., 16–19 years). Notably, investigations assessing responses to match-play in academy rugby union [14,16] or rugby league [6] players remain limited. Differing match demands [1,17], and differences in certain physical capabilities associated with specific age groups (i.e., reduced fitness levels and maximal strength) [18,19] appear to influence post-match recovery responses [1,6]. For this reason, there remains a need for practitioners to understand the magnitude and time-scale of post-match responses in academy players as this is likely to affect the implementation of recovery strategies and training regimes in the post-match period. This statement is especially true given that professional academy players often have additional commitments outside of their rugby careers in the form of school, college or additional employment, which may cause further restrictions and challenges when seeking to maximise recovery [20]. Collectively, differential post-match responses may be elicited in academy versus senior players when methods that incorporate greater ecological validity (i.e., the extent to which the findings are able to be generalised to real-life settings) [21] are employed. Therefore, in academy rugby league players, the aim of this study was to A) assess the within- and between-day reliability of neuromuscular and perceptual measures, before B) profiling the time-course of recovery of variables deemed reliable for 120 h post-match. The null hypothesis (H_0_) associated with part B of the study was that no differences would occur relative to baseline values after match-play. 

## 2. Materials and Methods

### 2.1. Experimental Overview

**Figure 1:**
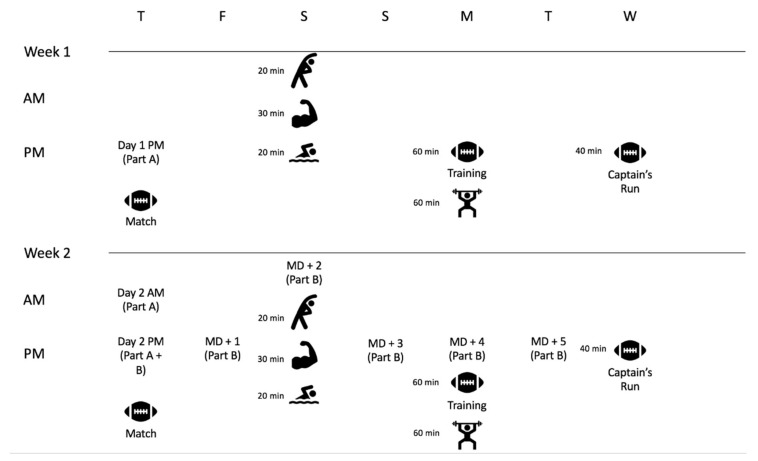
Study protocol. 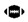 Match: Match-play; 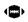 Training: The primary focus of this training session is development of specific skills and the tactical aspects of the game; 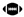 Captain’s run: The final training session leading up to the game. This session predominantly focuses on the tactical and game-specific elements of the game; 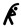: Static and dynamic stretching as well as full body foam rolling in order to restore range of motion and general movement function; 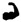: An upper-body hypertrophy-based training session; 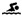: Pool session mostly taking place in the shallow end of the pool in which players perform a variety of dynamic movements (e.g., lunges, squats, calf raises, high knees); 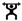: Individual gym-based program including a variety of full-body movements designed to improve strength, power and/or hypertrophy (e.g., bilateral squat variation, knee- and or hamstring-dominant hamstring exercises, lower-body unilateral exercises, horizontal and/or vertical push and pull exercises).

Figure 1 outlines the methods used in this study. In part A, this study assessed the reliability of isometric mid-thigh pull (IMTP), countermovement jump (CMJ), and wellness questionnaire measures in academy rugby league players. Within- (i.e., morning; AM vs afternoon; PM in week 2) and between-day (i.e., PM measures week 1 vs week 2) reliability was assessed during three visits over two days (i.e., week 1 day 1 PM, week 2 day 2 AM, week 2 day 2 PM). Each day was one week apart with the PM measure from the second day also serving as a baseline time-point for part B; occurring approximately 3 h before match-play commenced. Thereafter, in part B, the influence of match-play on variables deemed eligible (based on acceptable between-day reliability) was assessed for 120 h following a competitive rugby league match. After completion of the match, players were assessed at +24, +48, +72, +96 and +120 h.

### 2.2. Subjects

Following institutional ethical approval, 11 male rugby league players (age: 18 ± 1 years, mass: 92 ± 9 kg, stature: 1.83 ± 0.04 m, years spent in professional playing and training: 4 ± 1 years, three repetition maximum back squat: 141 ± 11 kg, three repetition maximum bench press: 93 ± 7 kg) from the same Super League academy (representing the highest tier of academy rugby league in England) volunteered to take part in the study. Players represented a range of positions but six played as forwards (three prop forwards, one back row forward, one loose forward and one hooker) with the remaining five players being backs (two wingers, two centers and one fullback). One player was unable to participate in visit one of the between-day component of part A; therefore, between-day comparisons, and part B responses represent ten players. Player absence was due to reasons unrelated to the study (i.e., injuries from previous matches, lack of availability for testing). Players were given full details of the study procedures and were informed of the risks and benefits of the study prior to the start of data collection. Retrospective power analyses indicated that >80% statistical power had been achieved for the statistically significant differences observed relative to baseline in CMJ jump height (JH). Players provided written informed consent (as well as parental/guardian consent where necessary; when players were <18 years of age). Although players had historically sustained a range of lower and upper body injuries, all were declared fit and free of illness or injury by the club’s medical staff at the time of testing.

### 2.3. Procedures

Upon arrival for testing, players first completed a wellness questionnaire, followed by a standard dynamic warm-up (including lunges, sweeps, hip openers, heel flicks, high knees and leg swings) and two submaximal attempts of the IMTP and the CMJ, before commencing the testing protocols. Match-play took place mid-season and locomotor activities were profiled using micro-electro-mechanical system (MEMS) devices. During the post-match period, players continued to participate in club activities (i.e., recovery strategies, training) as well as regular lifestyle commitments (e.g., college, school, work) as normal (Figure 1). Throughout the entire period of data collection, players were encouraged to maintain normal dietary intake, as advised by the club’s nutritionist. 

### 2.4. Subjective Wellness

Players completed a short wellness questionnaire adapted from McLean and colleagues [9] as per the supplementary materials (Table S1). This questionnaire, which players were accustomed to completing as part of routine monitoring practices at the club, required a rating of perceived fatigue, sleep quality, muscle soreness (separate ratings for upper- and lower-body), stress levels and mood on a five-point Likert scale. The aggregate sum of all six scores also provided a total wellness score. Lower values consistently indicated a negative response whilst higher values consistently indicated a positive response. Players completed the questionnaire separated from other individuals in order to minimise the influence of other players and/or coaching staff. The between-day reliability (coefficient of variation (CV): 7.1%) of this questionnaire has previously been reported in academy rugby union players during a non-training week [13]. 

### 2.5. Isometric Mid-Thigh Pull

In preparation for testing, participants took part in three habituation trials in the week prior to data collection. During the first habituation trial, players placed themselves in their preferred position whilst adhering to the prescribed guidelines as well as adhering to the range of joint angles (knee and hip angle of 120–135° and 140–150°, respectively) previously recommended [22]. Once the pulling position was established, starting positions were replicated between testing sessions to ensure repeatability of measures. Players were asked to stand on the force plate (type: FP4060-05-PT, dimensions: 600 mm × 400 mm, sampling: 1000 Hz, Bertec Corporation, Columbus, OH, USA) and to strap themselves to the bar using lifting straps (XXR Sports, Mitcham, UK) whilst achieving the correct body position that was previously determined during habituation. In this position, which replicated their second pull of the power clean, feet were roughly centred under the bar and hip-width apart. Knees were slightly flexed underneath and in front of the bar, whilst the torso was upright and shoulders retracted and depressed, above or slightly behind the vertical plane of the bar [22]. Using a goniometer (66fit, Spalding, UK), measurements were taken of both hip- and knee-angles to ensure players were in the correct position. Players were allowed minimal pre-tension to avoid any slack in the body prior to pull initiation [23]. In order to achieve optimal results, players were instructed to ‘push their feet into the floor’ and to ‘pull as hard and fast as possible’ [24]. Once stabilised (verified by watching the player and the force trace), a countdown was given, followed by a maximal effort of the IMTP. 

Visual inspection of the force-time curves during testing determined acceptability for inclusion. Trials were disregarded if an attempt included an unstable initial weighing period (i.e., clear fluctuation in the force-time data), if a clear countermovement (i.e., >50 N) took place prior to the pull, if peak force (PF) occurred at the end of the trial or if prior tension was applied before commencement of the pull (i.e., >50 N over body weight) [25]. Trials were also deemed invalid if PF was separated by >250 N between attempts or when a large change in body position was observed during the trial [25,26]. When incorrect trials took place, players were asked to repeat the test to ensure that each participant achieved three valid attempts. Players rested for a minimum of two min after each effort to ensure sufficient rest [27]. The IMTP testing was conducted as per the recommendations of Comfort et al. [26]. 

Based on the IMTP attempt during which PF was achieved, raw vertical force-time data were saved and exported into a Microsoft Excel file (Version 2019, Microsoft Corporation) which was later analysed. To identify the onset of the pull, this study used a threshold of five standard deviations (SD) of bodyweight, identified during one second of quiet standing immediately prior to commencing the pull (i.e., the weighing period) as per Reference [26]. The between-day reliability of PF, time-specific forces, and values elicited during IMTP time-bands have been found to be reliable (ICC ≥ 0.7, CV ≤ 15%) irrespective of body posture and barbell position [28].

### 2.6. Countermovement Jump

Players were instructed to stand on the force plate with their knees extended and feet in their preferred positions of slightly wider than shoulder-width apart whilst their hands remained on the hips. Following instruction to ‘jump as high and fast as they can,’ players dropped to a depth of their discretion and performed a jump for maximal height [29]. If, at any point during the jump, visual inspection deemed the hands to have come off the hips or legs being tucked in, the attempt was classified as invalid and the trial was repeated until three valid attempts were achieved. Players rested for a minimum of 60 s between trials [27]. 

Following a successful attempt, raw vertical force-time data were saved for the jump that elicited the greatest JH within a trial before being exported into a Microsoft Excel file which was later analysed. The start of the jump was identified as the time-point at which force deviated by five SD’s of bodyweight (measured during one second of quiet standing) [30]. Instances of take-off and touchdown were identified as the time-point whereby force deviated in excess of five times the SD during a 300 ms period of flight phase of the jump (i.e., when the platform was unloaded) [31]. This timeframe was taken at the end of the flight phase to avoid the unstable period of force-time data at the start of this phase. The between-day reliability of the CMJ has previously been reported in academy rugby union players during a non-training week (CV% < 5.0%) [13].

### 2.7. Match Load

A competitive home fixture took place during the mid-season (19:00 h kick off). Subjective internal match load was obtained by a session rating of perceived exertion (sRPE) within 30 min of the match finishing [32]. Players provided their individual score in isolation from others in order to minimise the influence of other players or coaches. The locomotive demands of the game were measured using portable MEMS units sampling at 10 Hz (Optimeye S5, Catapult Innovations, Melbourne, Australia). Units were worn in a pouch on the upper back of the playing shirt positioned between the shoulder blades. Additional gyroscopes, magnetometers and triaxial accelerometers sampling at 100 Hz captured information in relation to impact, accelerations, and decelerations. Devices were turned on just before the warm-up and turned off after the match. Following match completion, data were downloaded using proprietary software (Openfield Version 2.3.3, Catapult Innovations). Raw data files were trimmed on an individual player basis to ensure that only data pertaining to time spent on the pitch were exported for analysis. 

### 2.8. Statistical Analysis

For part A of the study, the within- and between-day reliability of variables was examined using mean changes between visits (assessed via paired samples t-tests), typical error (TE: SD of the differences score divided by √2), CV (typical error expressed as a percentage of the subject’s mean score), limits of agreement (LOA: mean bias ± 1.96 SD) and intraclass correlation (ICC: two-way mixed method, absolute agreement) values. Providing no significant differences existed, variables were deemed to have acceptable reliability in either component (i.e., on a within- or between-day basis) if both CV% was ≤10% [15] and ICC was ≥0.8 [33]. To evaluate the internal consistency of the wellness questionnaire, Cronbach’s Alpha (α) was also calculated [34]. The threshold for an acceptable α was set at >0.7 [35], whilst inter-item correlations were also considered. Only those variables that met the criteria for between-day reliability were eligible thereafter in part B of the study. For part B, initial assessments of normality were performed, before changes in post-match measures were analysed, using a repeated-measures analysis of variance (ANOVA) in statistical software (SPSS version 21, Chicago, IL, USA). Assumptions of sphericity were explored, and where necessary the Greenhouse-Geisser adjustment was used. If significant main effects were detected, data were compared using Bonferroni corrected pairwise comparisons. The criterion level of statistical significance was set at p ≤ 0.05. The magnitude of differences between all time-points was also expressed as a standardised mean difference (Cohen’s d effect size: ES). Classifications for ES were set as trivial (ES < 0.2), small (0.2 ≤ ES < 0.5), moderate (0.5 ≤ ES < 0.8) and large (ES ≥ 0.8) [36]. Data presented as mean ± SD unless otherwise stated.

## 3. Results

### 3.1. Isometric Mid-Thigh Pull Reliability

Reliability statistics for the IMTP are shown in Table 1 and Table 2. Acceptable within-day reliability was observed for PF, and force at 30 (F30), 150 (F150), 200 (F200), and 250 (F250) ms (CV%: 3.67–9.76%, ICC: 0.83–0.93). Acceptable between-day reliability values were observed for F200, F250 and PF (CV%: 4.34–8.62%; ICC: 0.87–0.92). Although no significant differences existed between repeated measurements, no other variables demonstrated acceptable reliability on either a within- or between-day basis.

### 3.2. Countermovement Jump Reliability

Reliability statistics for the CMJ are shown in Table 3 and Table 4. All variables, except for peak power (PP), relative PP and velocity at take-off (VTO), which were omitted due to the presence of significant differences between trials, showed acceptable levels of within-day reliability (CV%: 3.03–7.34%, ICC: 0.82–0.98). Six variables (i.e., flight-time (FT), PF, PP, relative PP, VTO, and JH) met the thresholds for acceptable between-day reliability (CV%: 2.56–6.79%; ICC: 0.83–0.91). The remaining five variables (i.e., movement-time (MT), FT:MT ratio, relative PF, time to PF, time to PP) did not meet the criteria for between-day reliability.

### 3.3. Subjective Wellness Reliability

Reliability statistics for the wellness questionnaire are shown in Table 5 and Table 6. Whilst some individual components of the questionnaire (i.e., sleep quality, lower body soreness, mood and total wellness) met the criteria of within-day reliability (CV%: 7.66–9.52%; ICC: 0.83–0.96), acceptable levels for between-day reliability were only found in the total wellness score (CV%: 7.05%; ICC: 0.90). The additional measure of Cronbach’s Alpha resulted in a value of α = 0.89, meaning that acceptable internal consistency was achieved by the items in the wellness questionnaire. Inter-item correlations are shown in Table 7.

### 3.4. Eligibility for Part B

Based on meeting the criteria for acceptable between-day reliability in Part A, the following variables were deemed eligible for part B: F200, F250 and PF in the IMTP; FT, PF, PP, relative PP, VTO and JH in the CMJ; and the total wellness score in the wellness questionnaire.

### 3.5. Match Demands

The average match load (i.e., RPE x time played) was 950 (±378) AU. Full locomotive match demands are presented in Table 8.

### 3.6. Isometric Mid-Thigh Pull Response

Match-play did not affect F200 (F(2,19) = 1.532, p = 0.240) or F250 (F(5,40) = 1.790, p= 0.137). Although match-play did show a significant time-effect for PF (F(5,40) = 2.782, p = 0.030), post-hoc measurements were unable to detect significance between time-points. Moderate (0.66) and large (0.90; 0.95) ES were observed at +24 h compared to baseline values for F200, F250 and PF, respectively. Trivial and small ES (≤0.37) were found at all other time-points thereafter compared to baseline values in PF, but moderate and large ES (≥0.67) were observed throughout the complete post-match period for F250. 

### 3.7. Countermovement Jump Response

**Figure 2:**
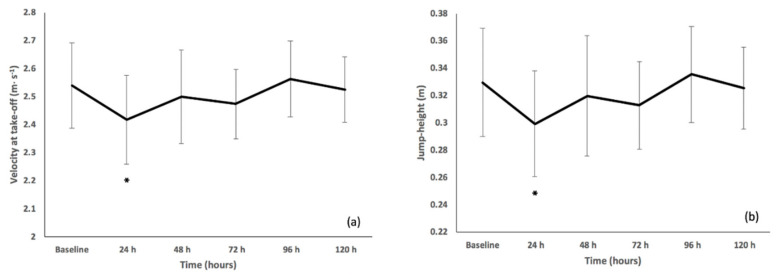
Mean (± standard deviation) countermovement jump velocity at take-off (**a**) and jump-height (**b**) before (baseline) and after (+24, +48, +72, +96, +120 h) rugby league match-play. * represents difference (p ≤ 0.05) relative to baseline.

Match-play influenced FT (F(5,40) = 5.638, p = 0.001) and although no changes relative to baseline were observed, values increased by 3.78% and 6.19% at +48 and +96 h, respectively, when compared to +24 h (0.502 s) values. Match-play also affected PF (F(2,19) = 4.627, p = 0.019) as values were increased by 11.84% at +96 h versus +24 h (2245 N). Although match-play influenced PP (F(5,40) = 4.992, p = 0.001) and relative PP (F(5,40) = 4.515, p = 0.002), no significant changes were detected between any of the time-points. Match-play influenced VTO (F(5,40) = 6.600, p < 0.001) and JH (F(5,40) = 6.527, p < 0.001) as values were decreased at +24 h compared to baseline (Figure 2a,b). Moderate and large ES (≥0.63) were reported at +24 h for all variables compared to baseline values. Trivial and small ES (≤0.41) compared to baseline values were then reported at +48 h for all variables except PP in which a moderate ES (0.70) existed. 

### 3.8. Wellness Response

The total wellness score was found to be influenced by match-play (F(5,40) = 5.962, p < 0.001). Although no post-match changes were found relative to baseline (23.55 points), values at +24 h were reduced by 8.99% versus +72 h values (21.00 points, p = 0.010). Large ES (0.86) compared to baseline values were reported at +24 h whilst moderate ES (≥0.56) were evident at +48 and +72 h.

## 4. Discussion

In professional academy rugby league players, the aims of this study were to assess the reliability of neuromuscular and wellness measures (part A) and to profile the time-course of such responses following match-play (part B). Acceptable within- and between-day reliability (i.e., no between-trial differences and CV% ≤ 10% and ICC ≥ 0.8) was achieved by F200, F250 and PF in the IMTP. Most CMJ variables demonstrated acceptable within-day reliability, whilst FT, PF, PP, relative PP, VTO and JH exhibited acceptable between-day reliability. From the wellness questionnaire, only the accumulated total wellness score met the threshold for between-day reliability, whereas four individual components of the wellness questionnaire (i.e., sleep quality, general lower body soreness, mood, total wellness) produced acceptable within-day reliability. The variables demonstrating acceptable between-day reliability were then eligible for use in part B of the study where match-play did not elicit statistically significant post hoc differences relative to baseline values for IMTP performance or total wellness. However, VTO and JH in the CMJ were depressed at +24 h versus baseline. Collectively, these findings indicate that the reliability of specific variables may differ when assessed on a within- or between-day basis. Similarly, the magnitude of the post-match response appeared to depend on the assessment and variables used. Such findings warrant consideration by practitioners when considering the type of measurements to be used in practice – especially when normal recovery, lifestyle and training activities are implemented by academy rugby league players in the post-match period.

Existing research indicated high within- and between-day reliability for IMTP forces elicited at earlier time-points (i.e., F30, F50, F90) in a variety of sporting populations [37,38]. These results are not reflected in the current study where force production at 30, 50, and 100 ms generally did not meet acceptable reliability thresholds. As dynamic tasks such as sprinting typically involve ground-contact times of between 50 and 250 ms [39], exposures to tasks that involve force production within <50 ms are limited in team sport players. It is plausible that this fact may explain the limited reliability of the F30 and F50 values in the present study. Across different sporting populations, the highest levels of reliability are typically found in forces produced at 200 and 250 ms and in PF [37]; findings which are in agreement with the results of the present study. 

Those CMJ variables demonstrating acceptable levels of between-day reliability (i.e., FT, PF, PP, relative PP, VTO and JH) are consistent across a number of sporting populations [15,29]. Time-related variables such as time to PF, time to PP, MT and consequently the FT:MT ratio did not meet the threshold for acceptable between-day reliability in the present study; findings which partly reflect those of previous research [29,40]. As the present study did not control for CMJ depth, players may have adopted an altered jump strategy when seeking to maximise jump height on each attempt [41]; especially in part B of the study. Allowing players to implement their preferred jump strategy may have inconsistently influenced displacement of their centre of mass during the eccentric and concentric phases across different jumps [41]. As a result, time-related variables may have been influenced by modification of the time spent in the eccentric and concentric phases of the movement with a view to maintaining the primary instruction of the jump, being to achieve maximal height. 

The monitoring questionnaire used here observed comparable reliability data to a similar questionnaire (i.e., one in which a 1–10 rating is required on soreness across a variety of sites), which was completed throughout the season by elite Australian Rules Football players [42]. Although comparable reliability (i.e., 7.1%) has been reported in a study of academy rugby union players [13], such scores may have reflected the absence of any physical activity undertaken between testing days. Akin to the methods of Montgomery and Hopkins [42], the present study was carried out whilst regular training activities were performed; a methodological issue that may influence different elements of the wellness questionnaire. Nevertheless, as the test-retest reliability of this type of questionnaire may be questioned when used in more ecologically valid scenarios (i.e., including regular training activities) [43], the current study may provide a more accurate representation of its within- and between-day reliability during the in-season period, and thus have implications for practitioners using such methods in similar scenarios. Notably, contrary to previous research [43], the internal consistency of the questionnaire (calculated via Cronbach’s Alpha) was deemed acceptable in the present study; a finding which may reflect the absence of negative values for inter-item correlations given that each question was aligned directionally (i.e., negative responses were always categorised as lower numerical values). 

Whilst responses to rugby match-play have been profiled using different measures, such as a CMJ [4,7,11], a plyometric push-up [14], and an adductor squeeze test [16], the present study is amongst the first to profile the effects of match-play on IMTP responses [44]. Although match-play did not influence PF during the IMTP, a large ES (0.95) was reported at +24 h following match-play compared to baseline measures, whilst small and trivial ES were observed thereafter. No significant changes were observed in F200 or F250 following match-play, but a large ES (0.9) in F250 was reported at +24 h versus baseline measures, whilst moderate and large ES (≥0.67) were still evident throughout the full post-match period. Prolonged perturbations (based on ES values) seen in some (i.e., F250), but not other (i.e., PF) variables suggest that maximal force production may be less sensitive to the influence of match-play when compared to those measures that include a velocity-component. This finding supports observations following Australian Rules Football match-play, in which the rate of force development was found to be more sensitive to the recovery of neuromuscular function than PF [44]. When performing sporting actions such as sprinting, jumping and changing direction, ground contact occurs in time intervals between 50–250 ms, therefore it may be more important to apply force quickly as opposed to producing maximal force [45]. Any reductions in F250 occurring post-match could have implications on athletic performance throughout the training week. 

Jump performance was reduced at +24 h following match-play, as indicated by significant differences (p ≤ 0.039) and large ES (≥1.44) in VTO and JH as well as moderate to large ES (≥0.63) compared to baseline values in FT, PP and PF. Small or trivial ES (≤0.41) were reported at +48 h after match-play compared to baseline values in FT, PF, VTO and JH, whilst ES observed in PP were still moderate (0.7) at this time-point. Accordingly, when using the CMJ to profile post-match responses, the magnitude of change may differ according to the variable selected; implications which could influence the interpretation of data derived, and thus prescription of training thereafter. Notably, a delayed recovery of PP compared to PF has previously been reported [8], with the present study lending some support to this observation (based on ES values). As the nature of rugby league includes a large frequency of sprinting, jumping and high-speed changes of direction, there is a large reliance on the ability to produce force rapidly [8]. For this reason, and because of its increased sensitivity to match-play, it may be more appropriate for practitioners to assess the velocity-components of CMJ testing rather than the force-components when seeking to profile post-exercise responses. Recovery of CMJ performance in this study was comparable to changes reported following competitive matches in academy rugby players [6,14]. However, prolonged reductions of larger magnitude were reported following competitive matches in senior players [4,5,7,11], which may be the result of differing peak movement and collision demands in this age group [2,46]. 

Even though match-play did not affect total wellness, large and moderate ES were found at +24 (0.86) and +48 h (0.76) compared to baseline measures, respectively. Disturbances in wellness in this study were similar to responses observed following competitive rugby matches in both senior and academy players [4,5,14], in which perturbations were present for up to +48 h. Even though acceptable internal consistency was found in the questionnaire, between-day reliability criteria were only met by total wellness. A more expansive scale (i.e., 0–10 or 0–100) may be useful to improve the reliability of all questions in this tool and enhance its practical application [47].

## 5. Conclusions

In conclusion, this study observed acceptable within- and between-day reliability in a variety of variables of the IMTP (i.e., F200, F250 and PF) and the CMJ (i.e., FT, PF, PP, relative PP, VTO and JH). Independent components of the wellness questionnaire should be interpreted with caution as acceptable between-day reliability was reported in total wellness only. Although match-play did not elicit significant post hoc differences for the majority of variables analysed (excluding VTO and JH), a large ES was observed in the post-match period for most variables (i.e., F200, F250 and PF of the IMTP, FT, PP of the CMJ and in the total wellness score) when compared to baseline measures. These results indicate that the magnitude and time-course of post-match responses may differ depending on the test and individual variables used. To avoid underestimation of the post-match response, it may be worthwhile to assess both objective (i.e., indices of neuromuscular fatigue) and subjective (i.e., total wellness) measures post-match-play. 

When taking IMTP measurements, practitioners working in rugby league are recommended to use F200, F250 and PF over forces elicited at earlier time-points due to the higher levels of within- and between-day reliability demonstrated in the present study. Likewise, because of its increased sensitivity to match-play, as well as the importance of rapid force application in sport, practitioners may consider the use of F250 over PF when profiling post-exercise responses. For the CMJ, analysis of variables such as FT, PF, PP, relative PP, VTO and JH may be preferred over a variety of other variables as a result of their greater between-day reliability. Assessing the velocity components of the CMJ may also assist in the interpretation of post-match responses. As individual components of the questionnaire lacked acceptable levels of between-day reliability, the use of total wellness is recommended when profiling post-exercise responses; especially given that this was the only element meeting the criteria for between-day reliability in this study. Collectively, post-match responses require at least 48 h to recover in academy rugby league players. During this time, practitioners are recommended to implement a variety of recovery strategies as this is likely to facilitate a quicker recovery of neuromuscular function and subjective wellness. Strenuous physical activity should be avoided in this time-period as this could prolong a return to baseline values.

## Figures and Tables

**Table 1:** Mean (± standard deviation) responses and the within-day reliability statistics for the isometric mid-thigh pull (*n* = 11).

Variable	Timing		Mean Change	TE (95% CI)	ICC (95% CI)	CV (95% CI)	LoA (95% CI)	Acceptable Reliability?
	Week 2 AM	Week 2 PM						
F30 (N)	1027.28 (71.72)	1053.19 (88.34)	25.91	42.27 (29.54, 74.18)	0.83 (0.40, 0.95)	3.91 (2.71, 6.96)	−143.08 (−244.05, −97.15) to 91.26 (45.33, 192.23)	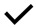
F50 (N)	1107.71 (110.67)	1146.77 (158.21)	39.06	91.89 (64.20, 161.26)	0.71 (−0.04, 0.92)	7.91 (5.46, 14.30)	−293.76 (−513.24, −193.92) to 215.64 (115.80, 435.12)	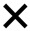
F100 (N)	1365.07 (242.26)	1420.24 (314.18)	55.16	174.83 (122.15, 306.81)	0.77 (0.14, 0.94)	11.58 (7.96, 21.20)	−539.76 (−957.34, −349.80) to 429.43 (239, 47, 847.01)	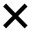
F150 (N)	1623.64 (321.37)	1670.13 (344.87)	46.49	159.27 (111.28, 279.50)	0.88 (0.55, 0.97)	9.76 (6.73, 17.76)	−487.96 (−868.38, −314.90) to 394.98 (221.92, 775.40)	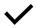
F200 (N)	1858.82 (349.72)	1901.68 (351.99)	42.86	154.58 (108.01, 271.28)	0.90 (0.63, 0.97)	8.41 (5.81, 15.23)	−471.33 (−840.56, −303.37) to 385.62 (217.66, 754.85)	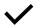
F250 (N)	2022.65 (331.77)	2075.84 (326.60)	53.19	145.61 (101.74, 255.53)	0.89 (0.62, 0.97)	7.17 (4.96, 12.93)	−456.79 (−804.58, −298.58) to 350.41 (192.20, 698.20)	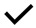
PF (N)	2577.09 (279.00)	2628.41 (264.70)	51.32	97.36 (68.03, 170.87)	0.93 (0.74, 0.98)	3.67 (2.55, 6.53)	−321.20 (−553.754, −215.40) to 218.56 (112.77, 451.12)	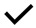

AM: Morning; CI: Confidence interval; CV%: Coefficient of variation; F30: Force at 30 ms; F50: Force at 50 ms; F100: Force at 100 ms; F150: Force at 150 ms; F200: Force at 200 ms; F250: Force at 250 ms; ICC: Intraclass correlation coefficient; LoA: Limits of agreement; PF: Peak force; PM: Afternoon; TE: Typical error. Acceptable reliability was defined as no between-trial differences and CV ≤ 10% and ICC ≥ 0.8.

**Table 2:** Mean (± standard deviation) responses and the between-day reliability statistics for the isometric mid-thigh pull (*n* = 10).

Variable	Timing		Mean Change	TE (95% CI)	ICC (95% CI)	CV (95% CI)	LoA (95% CI)	Acceptable Reliability?
	Week 1 PM	Week 2 PM						
F30 (N)	1040.80 (59.00)	1051.26 (92.87)	10.46	61.40 (42.24, 112.10)	0.57 (−0.95, 0.90)	6.07 (4.14, 11.36)	−180.65 (−340.38, −111.40) to 159.75 (90.49, 319.47)	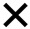
F50 (N)	1127.46 (94.04)	1150.87 (166.15)	23.41	109.39 (75.24, 199.69)	0.53 (−1.10, 0.89)	9.86 (6.68, 18.73)	−326.61 (−611.15, −203.24) to 279.79 (156.42, 564.32)	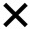
F100 (N)	1404.13 (215.80)	1429.48 (329.59)	25.35	200.08 (137.62, 365.26)	0.67 (−0.45, 0.92)	14.20 (9.56, 27.43)	−579.93 (−1100.37, −354.27) to 529.23 (303.57, 1049.67)	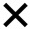
F150 (N)	1677.54 (281.51)	1670.28 (363.52)	7.26	170.75 (117.45, 311.72)	0.85 (0.38, 0.96)	10.91 (7.38, 20.82)	−466.03 (−910.18, −273.44) to 480.56 (287.97, 924.71)	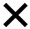
F200 (N)	1921.20 (297.20)	1895.69 (370.44)	25.51	154.48 (106.26, 282.02)	0.89 (0.55, 0.97)	8.62 (5.58, 16.29)	−402.68 (−804.52, −228.45) to 453.71 (279.48, 855.55)	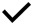 *
F250 (N)	2078.98 (288.99)	2073.48 (344.17)	5.50	158.55 (109.06, 289.45)	0.87 (0.45, 0.97)	8.01 (5.44, 15.11)	−433.98 (−846.40, −255.16) to 444.98 (266.15, 857.40)	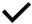 *
PF (N)	2593.47 (288.46)	2627.58 (279.00)	34.11	112.46 (82.02, 185.01)	0.92 (0.68, 0.98)	4.34 (3.15, 7.24)	−345.82 (−638.34, −218.98) to 277.61 (150.78, 570.14)	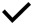 *

CI: Confidence interval; CV%: Coefficient of variation; F30: Force at 30 ms; F50: Force at 50 ms; F100: Force at 100 ms; F150: Force at 150 ms; F200: Force at 200 ms; F250: Force at 250 ms; ICC: Intraclass correlation coefficient; LoA: Limits of agreement; PF: Peak force; PM: Afternoon; TE: Typical error. Acceptable reliability was defined as no between-trial differences and CV ≤ 10% and ICC ≥ 0.8. * Variable met the criteria for between-day reliability and was therefore eligible for Part B of the study.

**Table 3:** Mean (± standard deviation) responses and the within-day reliability statistics for the countermovement jump (*n* = 11).

Variable	Timing		Mean Change	TE (95% CI)	ICC (95% CI)	CV (95% CI)	LoA (95% CI)	Acceptable Reliability?
	Week 2 AM	Week 2 PM						
MT (s)	0.74 (0.12)	0.71 (0.10)	0.03	0.04 (0.03, 0.07)	0.91 (0.64, 0.98)	5.97 (4.07, 11.17)	−0.09 (−0.20, −0.04) to 0.15 (0.10, 0.26)	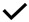
FT (s)	0.51 (0.03)	0.52 (0.04)	0.01	0.02 (0.01, 0.03)	0.88 (0.45, 0.97)	3.03 (2.07, 5.60)	−0.06 (−0.10, −0.04) to 0.03 (0.01, 0.07)	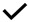
MT:FT ratio	0.69 (0.10)	0.74 (0.10)	0.05	0.05 (0.03, 0.09)	0.82 (0. 26, 0.96)	7.34 (4.99, 13.80)	−0.19 (−0.32, −0.13) to 0.09 (0.31, 0.22)	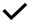
PF (N)	2362.00 (367.12)	2411.32 (369.62)	49.32	77.33 (53.19, 141.18)	0.98 (0.90, 0.99)	3.15 (2.15, 5.82)	−263.67 (−464.82, −176.45) to 165.03 (77.81, 366.18)	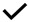
Relative PF (N·kg^−1^ BW)	25.54 (2.85)	25.88 (2.98)	0.34	0.89 (0.61, 1.62)	0.95 (0.82, 0.98)	3.34 (2.29, 6.19)	−2.79 (−5.10, −1.80) to 2.12 (1.12, 4.42)	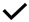
Time to PF (s)	0.55 (0.10)	0.52 (0.08)	0.03	0.04 (0.02, 0.07)	0.89 (0.54, 0.97)	7.09 (4.83, 13.33)	−0.07 (−0.16, −0.03) to 0.13 (0.09, 0.23)	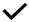
PP (W)	4644.38 (453.47)	4939.47 ** (507.11)	295.09	132.89 (91.41, 242.61)	0.88 (−0.13, 0.98)	2.75 (1.88, 5.07)	−263.67 (−464.82, −176.45) to 165.03 (77.81, 366.18)	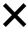
Relative PP (W·kg^−1^ BW)	50.42 (3.78)	53.22 (4.73) **	2.80	1.61 (1.11, 2.93)	0.84 (−0.091, 0.97)	2.94 (2.02, 5.44)	−7.25 (−11.43, −5.44) to 1.66 (−0.16, 5.84)	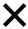
Time to PP (s)	0.68 (0.12)	0.64 (0.10)	0.04	0.04 (0.03, 0.08)	0.92 (0.67, 0.98)	6.29 (4.29, 11.79)	−0.08 (−0.19, −0.04) to 0.15 (0.10, 0.26)	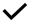
VTO (m·s^−1^)	2.46 (0.16)	2.54 (0.18) **	0.08	0.06 (0.04, 0.12)	0.87 (0.24, 0.97)	2.58 (1.77, 4.77)	−0.26 (−0.43, −0.19) to 0.09 (0.02, 0.26)	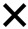
JH (m)	0.31 (0.04)	0.33 (0.05)	0.02	0.02 (0.01, 0.03)	0.89 (0.62, 0.97)	5.23 (3.57, 9.76)	−0.07 (−0.11, −0.05) to 0.02 (0.01, 0.07)	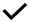

AM: Morning; BW: Body weight; CI: Confidence interval; CV%: Coefficient of variation; F30: Force at 30 ms; F50: Force at 50 ms; F100: Force at 100 ms; F150: Force at 150 ms; F200: Force at 200 ms; F250: Force at 250 ms; FT: Flight time; ICC: Intraclass correlation coefficient; JH: Jump height; LoA: Limits of agreement; MT: Movement time; PF: Peak force; PM: Afternoon; PP: Peak power; TE: Typical error; VTO: Velocity at take-off; **: Significantly different (p ≤ 0.05) from week 2 AM. Acceptable reliability was defined as no between-trial differences and CV ≤ 10% and ICC ≥ 0.8.

**Table 4:** Mean (± standard deviation) responses and the between-day reliability statistics for the countermovement jump (*n*=10).

Variable	Timing		Mean Change	TE (95% CI)	ICC (95% CI)	CV (95% CI)	LoA (95% CI)	Acceptable Reliability?
	Week 1 PM	Week 2 PM						
MT (s)	0.75 (0.10)	0.71 (0.11)	0.04	0.08 (0.05, 0.15)	0.63 (−0.42, 0.91)	10.5 (6.97, 21.07)	−0.16 (−0.38, −0.08) to 0.26 (0.17, 0.47)	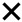
FT (s)	0.52 (0.03)	0.53 (0.04)	0.01	0.02 (0.01, 0.03)	0.89 (0.57, 0.98)	3.08 (2.07, 5.98)	−0.05 (−0.10, −0.04) to 0.03 (0.02, 0.08)	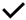 *
MT:FT ratio	0.70 (0.09)	0.76 (0.10)	0.06	0.07 (0.05, 0.13)	0.59 (−0.36, 0.90)	10.11 (6.72, 20.26)	−0.25 (−0.45, −0.17) to 0.13 (0.05, 0.32)	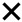
PF (N)	2346.17 (301.12)	2437.74 (381.90)	91.57	146.96 (99.26, 281.53)	0.89 (0.56, 0.98)	6.79 (4.54, 13.41)	−498.91 (−920.82, −326.24) to 315.77 (143.10, 737.67)	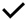 *
Relative PF (N·kg^−1^ BW)	25.43 (2.19)	26.23 (2.93)	0.80	1.71 (1.15, 3.28)	0.72 (−0.15, 0.94)	7.02 (4.69, 13.88)	−5.54 (−10.45, −3.53) to 3.94 (1.93, 8.84)	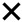
Time to PF (s)	0.58 (0.11)	0.51 (0.08)**	0.07	0.07 (0.04, 0.13)	0.60 (−0.29, 0.91)	11.19 (7.43, 22.54)	−0.11 (−0.30, −0.03) to 0.26 (0.18, 0.44)	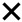
PP (W)	4898.03 (465.94)	5020.36 (464.44)	122.33	208.63 (140.92, 399.68)	0.88 (0.52, 0.97)	4.56 (3.05, 8.91)	−700.61 (−1299.58, −455.48) to 455.95 (210.82, 1054.92)	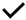 *
Relative PP (W·kg^−1^ BW)	53.30 (5.01)	54.25 (3.66)	0.95	2.38 (1.61, 4.55)	0.83 (0.29, 0.96)	4.73 (3.17, 9.25)	−7.54 (−14.36, −4.74) to 5.64 (2.85, 12.46)	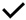 *
Time to PP (s)	0.69 (0.10)	0.64 (0.11)	0.05	0.08 (0.05, 0.15)	0.63 (−0.42, 0.91)	11.59 (7.69, 23.39)	−0.17 (−0.39, −0.08) to 0.26 (0.17, 0.48)	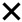
VTO (m·s^−1^)	2.54 (0.15)	2.57 (0.17)	0.03	0.06 (0.04, 0.12)	0.91 (0.64, 0.98)	2.56 (1.72, 4.97)	−0.21 (−0.39, −0.13) to 0.15 (0.07, 0.33)	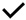 *
JH (m)	0.33 (0.04)	0.34 (0.04)	0.01	0.02 (0.01, 0.03)	0.91 (0.65, 0.98)	5.19 (3.48, 10.18)	−0.05 (−0.10, −0.03) to 0.04 (0.02, 0.09)	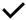 *

BW: Body weight; CI: Confidence interval; CV%: Coefficient of variation; F30: Force at 30 ms; F50: Force at 50 ms; F100: Force at 100 ms; F150: Force at 150 ms; F200: Force at 200 ms; F250: Force at 250 ms; FT: Flight time; ICC: Intraclass correlation coefficient; JH: Jump height; LoA: Limits of agreement; MT: Movement time; PF: Peak force; PM: Afternoon; PP: Peak power; TE: Typical error; VTO: Velocity at take-off. * Variable met the criteria for between-day reliability and was therefore eligible for Part B of the study; **: Significantly different (p ≤ 0.05) from week 1 PM; Acceptable reliability was defined as no between-trial differences and CV ≤ 10% and ICC ≥ 0.8.

**Table 5:** Mean (± standard deviation) responses and the within-day reliability statistics for the wellness questionnaire (*n* = 11).

Variable	Timing		Mean Change	TE (95% CI)	ICC (95% CI)	CV (95% CI)	LoA (95% CI)	Acceptable Reliability?
	Week 2 AM	Week 2 PM						
Fatigue	3.36 (0.81)	3.91 (0.83)	0.55	0.73 (0.51, 1.29)	0.30 (−0.93, 0.79)	24.85 (16.77, 47.62)	−2.58 (−4.33, −1.78) to 1.48 (0.69, 3.23)	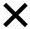
Sleep quality	3.73 (0.79)	3.91 (0.83)	0.18	0.29 (0.20, 0.50)	0.93 (0.74, 0.98)	7.66 (5.29, 13.82)	−0.97 (−1.66, −0.66) to 0.61 (0.30, 1.29)	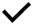
General upper body soreness	3.18 (0.60)	3.64 (0.81) **	0.45	0.37 (0.26, 0.65)	0.77 (0.04, 0.94)	10.77 (7.41, 19.66)	−1.48 (−2.36, −1.08) to 0.57 (0.17, 1.45)	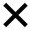
General lower body soreness	3.00 (1.10)	3.00 (1.10)	0.00	0.32 (0.22, 0.55)	0.96 (0.85, 0.99)	9.52 (6.56, 17.31)	−0.88 (−1.63, −0.53) to 0.88 (0.53, 1.63)	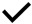
Stress level	4.09 (0.54)	3.82 (0.87)	0.27	0.56 (0.39, 0.98)	0.58 (−0.45, 0.88)	19.61 (13.33, 36.92)	−1.27 (−2.60, −0.66) to 1.81 (1.20, 3.14)	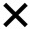
Mood	4.27 (0.65)	4.27 (0.47)	0.00	0.32 (0.22, 0.55)	0.83 (0.33, 0.95)	8.47 (5.84, 15.33)	−0.88 (−1.63, −0.53) to 0.88 (0.53, 1.63)	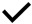
Total wellness score	21.64 (2.98)	22.55 (3.78)	0.91	1.80 (1.26, 3.16)	0.83 (0.42, 0.95)	9.20 (6.35, 16.71)	−5.90 (−10.21, −3.95) to 4.08 (2.13, 8.39)	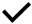

AM; Morning; CI: Confidence interval; CV%: Coefficient of variation; ICC: Intraclass correlation coefficient; LoA: Limits of agreement; PM: Afternoon; TE: Typical error; **: Significantly different (p ≤ 0.05) from week 2 AM. Acceptable reliability was defined as no between-trial differences and CV ≤ 10% and ICC ≥ 0.8.

**Table 6:** Mean (± standard deviation) responses and the between-day reliability statistics for the wellness questionnaire (n = 10).

Variable	Timing		Mean Change	TE (95% CI)	ICC (95% CI)	CV (95% CI)	LoA (95% CI)	Acceptable Reliability?
	Week 1 PM	Week 2 PM						
Fatigue	3.30 (0.95)	3.80 (0.79)	0.50	0.60 (0.41, 1.10)	0.64 (−0.18, 0.91)	20.36 (13.59, 40.25)	−2.17 (−3.73, −1.49) to 1.17 (0.49, 2.73)	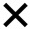
Sleep quality	3.80 (0.42)	3.90 (0.88)	0.10	0.40 (0.28, 0.73)	0.81 (0.21, 0.95)	12.87 (8.68, 24.73)	−1.21 (−2.26, −0.76) to 1.01 (0.56, 2.06)	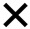
General upper body soreness	3.40 (0.52)	3.60 (0.84)	0.20	0.65 (0.45, 1.19)	0.25 (−2.40, 0.82)	22.86 (15.21, 45.63)	−2.00 (−3.69, −1.27) to 1.60 (0.87, 3.29)	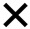
General lower body soreness	3.00 (1.05)	3.10 (1.10)	0.10	0.62 (0.43, 1.13)	0.82 (0.23, 0.96)	23.14 (15.39, 46.22)	−1.82 (−3.43, −1.12) to 1.62 (0.92, 3.23)	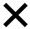
Stress level	3.90 (0.74)	3.80 (0.92)	0.10	0.40 (0.28, 0.73)	0.88 (0.51, 0.97)	14.11 (9.50, 27.24)	−1.01 (−2.06, −0.56) to 1.21 (0.76, 2.26)	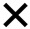
Mood	4.10 (0.57)	4.30 (0.48)	0.20	0.30 (0.21, 0.54)	0.79 (0.24, 0.95)	7.99 (5.43, 15.07)	−1.03 (−1.80, −0.69) to 0.63 (0.29, 1.40)	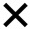
Total wellness score	21.50 (3.31)	22.50 (3.98)	1.00	1.53 (1.05, 2.97)	0.90 (0.60, 0.97)	7.05 (4.80, 13.24)	−5.23 (−9.21, −3.51) to 3.23 (1.51, 7.21)	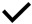 *

CI: Confidence interval; CV%: Coefficient of variation; ICC: Intraclass correlation coefficient; LoA: Limits of agreement; PM: Afternoon; TE: Typical error. Acceptable reliability was defined as no between-trial differences and CV ≤ 10% and ICC ≥ 0.8. * Variable met the criteria for between-day reliability and was therefore eligible for Part B of the study.

**Table 7:** Subjective wellness inter-item correlation matrix.

Questionnaire Item	Sleep Quality	Upper Body Soreness	Lower Body Soreness	Stress Level	Mood
Fatigue	0.29	0.80	0.74	0.71	0.67
Sleep Quality	-	0.69	0.48	0.21	0.22
Upper Body Soreness	-	-	0.85	0.56	0.67
Lower Body Soreness	-	-	-	0.83	0.81
Stress Level	-	-	-	-	0.71
Mood	-	-	-	-	-

**Table 8:** Mean (± standard deviation) locomotive match demands (n = 10).

Timing	Duration (min)	Total Distance		High-Speed (≥5.5 m⋅s^−1^) Running (m)	Player Load (AU)	Repeated High-Intensity Efforts (n)
		Absolute (m)	Relative (m⋅min^−1^)			
Warm-Up	24:21 (00:00)	1648 (230)	68 (9)	50 (49)	174 (21)	9 (2)
First Half	31:36 (14:35)	2756 (1215)	91 (12)	111 (86)	275 (119)	15 (6)
Second Half	37:33 (13:23)	2938 (1046)	80 (10)	58 (46)	283 (99)	15 (5)

AU: Arbitrary units.

## Supplementary Materials

The following are available online at https://www.mdpi.com/2075-4663/8/5/73/s1, Table S1: Wellness questionnaire.

## References

[1] Johnston R.D., Gabbett T.J., Jenkins D.G. (2015). Influence of playing standard and physical fitness on activity profiles and post-match fatigue during intensified junior rugby league competition. Sports Med. Open.

[2] Johnston R.D., Weaving D., Hulin B.T., Till K., Jones B., Duthie G. (2019). Peak movement and collision demands of professional rugby league competition. J. Sports Sci..

[3] Weaving D., Sawczuk T., Williams S., Scott T., Till K., Beggs C., Johnston R.D., Jones B. (2019). The peak duration-specific locomotor demands and concurrent collision frequencies of European Super League rugby. J. Sports Sci..

[4] Oxendale C.L., Twist C., Daniels M., Highton J. (2016). The relationship between match-play characteristics of elite rugby league and indirect markers of muscle damage. Int. J. Sport Physiol. Perform..

[5] Twist C., Waldron M., Highton J., Burt D., Daniels M. (2012). Neuromuscular, biochemical and perceptual post-match fatigue in professional rugby league forwards and backs. J. Sports Sci..

[6] Johnston R.D., Gabbett T.J., Jenkins D.G., Hulin B.T. (2015). Influence of physical qualities on post-match fatigue in rugby league players. J. Sci. Med. Sport..

[7] McLellan C.P., Lovell D.I. (2012). Neuromuscular responses to impact and collision during elite rugby league match play. J. Strength Cond. Res..

[8] McLellan C.P., Lovell D.I., Gass G.C. (2011). Markers of postmatch fatigue in professional rugby league players. J. Strength Cond. Res..

[9] McLean B.D., Coutts A.J., Kelly V., McGuigan M.R., Cormack S.J. (2010). Neuromuscular, endocrine, and perceptual fatigue responses during different length between-match microcycles in professional rugby league players. Int. J. Sport Physiol. Perform..

[10] Kellmann M., Bertollo M., Bosquet L., Brink M., Coutts A.J., Duffield R., Erlacher D., Halson S.L., Hecksteden A., Heidari J. (2018). Recovery and performance in sport: Consensus statement. Int. J. Sport Physiol. Perform..

[11] West D.J., Finn C.V., Cunningham D.J., Shearer D.A., Jones M.R., Harrington B.J., Crewther B.T., Cook C.J., Kilduff L.P. (2014). Neuromuscular function, hormonal, and mood responses to a professional rugby union match. J. Strength Cond. Res..

[12] Aben H.G.J., Hills S.P., Cooke C.B., Davis D., Jones B., Russell M. (2020). Profiling the post-match recovery response in male rugby: A systematic review. J. Strength Cond. Res..

[13] Roe G., Darrall-Jones J., Till K., Phibbs P., Read D., Weakley J., Jones B. (2016). Between-days reliability and sensitivity of common fatigue measures in rugby players. Int. J. Sport Physiol. Perform..

[14] Roe G., Till K., Darrall-Jones J., Phibbs P., Weakley J., Read D., Jones B. (2016). Changes in markers of fatigue following a competitive match in elite academy rugby union players. S. Afr. J. Sports Med..

[15] Cormack S.J., Newton R.U., McGuigan M.R., Doyle T.L. (2008). Reliability of measures obtained during single and repeated countermovement jumps. Int. J. Sport Physiol. Perform..

[16] Roe G.A., Phibbs P.J., Till K., Jones B.L., Read D.B., Weakley J.J., Darrall-Jones J.D. (2016). Changes in adductor strength after competition in academy rugby union players. J. Strength Cond. Res..

[17] McLellan C.P., Lovell D.I. (2013). Performance analysis of professional, semiprofessional, and junior elite rugby league match-play using global positioning systems. J. Strength Cond. Res..

[18] Gabbett T.J. (2002). Physiological characteristics of junior and senior rugby league players. Br. J. Sports Med..

[19] Till K., Tester E., Jones B., Emmonds S., Fahey J., Cooke C. (2014). Anthropometric and physical characteristics of English academy rugby league players. J. Strength Cond. Res..

[20] Hendricks S., Till K., Weaving D., Powell A., Kemp S., Stokes K., Jones B. (2019). Training, match and non-rugby activities in elite male youth rugby union players in England. Int. J. Sports Sci. Coa..

[21] Lewkowicz D.J. (2001). The concept of ecological validity: What are its limitations and is it bad to be invalid?. Infancy.

[22] Beckham G.K., Sato K., Santana H.A., Mizuguchi S., Haff G.G., Stone M.H. (2018). Effect of body position on force production during the isometric midthigh pull. J. Strength Cond. Res..

[23] Mangine G.T., Hoffman J.R., Wang R., Gonzalez A.M., Townsend J.R., Wells A.J., Jajtner A.R., Beyer K.S., Boone C.H., Miramonti A.A. (2016). Resistance training intensity and volume affect changes in rate of force development in resistance-trained men. Eur. J. Appl. Physiol..

[24] Halperin I., Williams K.J., Martin D.T., Chapman D.W. (2016). The effects of attentional focusing instructions on force production during the isometric midthigh pull. J. Strength Cond. Res..

[25] Dos’Santos T., Jones P.A., Comfort P., Thomas C. (2017). Effect of Different Onset Thresholds on Isometric Midthigh Pull Force-Time Variables. J. Strength Cond. Res..

[26] Comfort P., Dos’ Santos T., Beckham G.K., Stone M.H., Guppy S.N., Haff G.G. (2019). Standardization and methodological considerations for the isometric midthigh pull. Strength Cond. J..

[27] Thomas C., Comfort P., Jones P.A., Dos’Santos T. (2017). A Comparison of isometric midthigh-pull strength, vertical jump, sprint speed, and change-of-direction speed in academy netball players. Int. J. Sport Physiol. Perform..

[28] Guppy S., Brady C., Kotani Y., Stone M., Medic N., Haff G. (2018). The effect of altering body posture and barbell position on the between-session reliability of force-time curve characteristics in the isometric mid-thigh pull. Sports.

[29] McMahon J.J., Murphy S., Rej S.J., Comfort P. (2017). Countermovement-jump-phase characteristics of senior and academy rugby league players. Int. J. Sport Physiol. Perform..

[30] West D.J., Owen N.J., Jones M.R., Bracken R.M., Cook C.J., Cunningham D.J., Shearer D.A., Finn C.V., Newton R.U., Crewther B.T. (2011). Relationships between force–time characteristics of the isometric midthigh pull and dynamic performance in professional rugby league players. J. Strength Cond. Res..

[31] Moir G.L. (2008). Three different methods of calculating vertical jump height from force platform data in men and women. Meas. Phys. Educ. Exerc. Sci..

[32] Borg G. (1998). Borg’s Perceived Exertion and Pain Scales.

[33] Comfort P., Jones P.A., McMahon J.J., Newton R. (2015). Effect of knee and trunk angle on kinetic variables during the isometric midthigh pull: Test–retest reliability. Int. J. Sport Physiol..

[34] Cronbach L.J. (1951). Coefficient alpha and the internal structure of tests. Psychometrika.

[35] Bland J.M., Altman D.G. (1997). Statistics notes: Cronbach’s alpha. Br. Med. J..

[36] Fritz C.O., Morris P.E., Richler J.J. (2012). Effect size estimates: Current use, calculations, and interpretation. J. Exp. Psychol. Gen..

[37] Haff G.G., Ruben R.P., Lider J., Twine C., Cormie P. (2015). A comparison of methods for determining the rate of force development during isometric midthigh clean pulls. J. Strength Cond. Res..

[38] Dos’ Santos T., Thomas C., Comfort P., McMahon J.J., Jones P.A., Oakley N.P., Young A.L. (2018). Between-session reliability of isometric midthigh pull kinetics and maximal power clean performance in male youth soccer players. J. Strength Cond. Res..

[39] Aagaard P., Simonsen E.B., Andersen J.L., Magnusson P., Dyhre-Poulsen P. (2002). Increased rate of force development and neural drive of human skeletal muscle following resistance training. J. Appl. Physiol..

[40] Hori N., Newton R.U., Kawamori N., McGuigan M.R., Kraemer W.J., Nosaka K. (2009). Reliability of performance measurements derived from ground reaction force data during countermovement jump and the influence of sampling frequency. J. Strength Cond. Res..

[41] McMahon J.J., Rej S.J., Comfort P. (2017). Sex differences in countermovement jump phase characteristics. Sports.

[42] Montgomery P.G., Hopkins W.G. (2013). The effects of game and training loads on perceptual responses of muscle soreness in Australian football. Int. J. Sport Physiol. Perform..

[43] Fitzpatrick J., Hicks K., Russell M., Hayes P. (2019). The reliability of potential fatigue-monitoring measures in elite youth soccer players. J. Strength Cond Res..

[44] Norris D., Joyce D., Siegler J., Clock J., Lovell R. (2019). Recovery of force–time characteristics after Australian rules football matches: Examining the utility of the isometric midthigh pull. Int. J. Sport Physiol. Perform..

[45] Dos’Santos T., Thomas C., Comfort P., McMahon J., Jones P. (2017). Relationships between isometric force-time characteristics and dynamic performance. Sports.

[46] Whitehead S., Till K., Weaving D., Dalton-Barron N., Ireton M., Jones B. (2018). The duration-specific peak average running speeds of European super league academy rugby league match-play. J. Strength Cond. Res..

[47] McLaren S.J., Smith A., Spears I.R., Weston M. (2017). A detailed quantification of differential ratings of perceived exertion during team-sport training. J. Sci. Med. Sport.

