# Supplementary material for: The Reliability of Neuromuscular and Perceptual Measures Used to Profile Recovery, and the Time-Course of Such Responses Following Academy Rugby League Match-Play
Source: Sports (Basel). 2020 May 22;8(5):73. doi: 10.3390/sports8050073 (PMC7281125; doi:10.3390/sports8050073)
Supplement: Supplementary file 1 [file sports-08-00073-s001.pdf]

sports

Supplementary Materials: The Reliability of Neuromuscular and Perceptual Measures used to profile Recovery, and the Time-Course of such Responses following Academy Rugby League Match-Play

Hendrickus G.J. Aben, Samuel P. Hills, Darren Higgens, Carlton B. Cooke, Danielle Davis, Ben Jones and Mark Russell *

Table S1. Wellness questionnaire

	1	2	3	4	5
Fatigue	Always tired	More tired than normal	Normal	Fresh	Very fresh
Sleep Quality	Insomnia	Restless sleep	Difficulty falling asleep	Good	Very restful
General Upper Body Muscle Soreness	Very Sore	Increase in soreness/tightness	Normal	Feeling good	Feeling great
General Lower Body Muscle Soreness	Very Sore	Increase in soreness/tightness	Normal	Feeling good	Feeling great
Stress Level	Highly stressed	Feeling stressed	Normal	Relaxed	Very relaxed
Mood	Highly annoyed/irritable/down	Aggrevated/short tempered	Less interested in others and/or activities than usual	A generally good mood	Very positive mood

© 2020 by the authors. Licensee MDPI, Basel, Switzerland. This article is an open access article distributed under the terms and conditions of the Creative Commons Attribution (CC BY) license (http://creativecommons.org/licenses/by/4.0/).
